# Testing the Protective Effects of Sulfobutylether-Βeta-Cyclodextrin (SBECD) and Sugammadex against Chlorpromazine-Induced Acute Toxicity in SH-SY5Y Cell Line and in NMRI Mice

**DOI:** 10.3390/pharmaceutics14091888

**Published:** 2022-09-07

**Authors:** Eszter Fliszár-Nyúl, Rita Csepregi, Gábor Benkovics, Lajos Szente, Miklós Poór

**Affiliations:** 1 Department of Pharmacology, Faculty of Pharmacy, University of Pécs, H-7624 Pécs, Hungary; 2 Lab-on-a-Chip Research Group, János Szentágothai Research Centre, University of Pécs, H-7624 Pécs, Hungary; 3 Department of Laboratory Medicine, Medical School, University of Pécs, H-7624 Pécs, Hungary; 4 CycloLab Cyclodextrin Research & Development Laboratory, Ltd., H-1097 Budapest, Hungary

**Keywords:** chlorpromazine, cyclodextrins, sulfobutylether-β-cyclodextrin, sugammadex, inclusion complexes, detoxification

## Abstract

Chlorpromazine (CPZ) is an antipsychotic drug which can cause several adverse effects and drug poisoning. Recent studies demonstrated that CPZ forms highly stable complexes with certain cyclodextrins (CDs) such as sulfobutylether-β-CD (SBECD) and sugammadex (SGD). Since there is no available antidote in CPZ intoxication, and considering the good tolerability of these CDs even if when administered parenterally, we aimed to investigate the protective effects of SBECD and SGD against CPZ-induced acute toxicity employing in vitro (SH-SY5Y neuroblastoma cells) and in vivo (zebrafish embryo) models. Our major findings and conclusions are the following: (1) both SBECD and SGD strongly relieved the cytotoxic effects of CPZ in SH-SY5Y cells. (2) SGD co-treatment did not affect or increase the CPZ-induced 24 h mortality in NMRI mice, while SBECD caused a protective effect in a dose-dependent fashion. (3) The binding constants of ligand–CD complexes and/or the in vitro protective effects of CDs can help to estimate the in vivo suitability of CDs as antidotes; however, some other factors can overwrite these predictions.

## 1. Introduction

**Figure 1:**
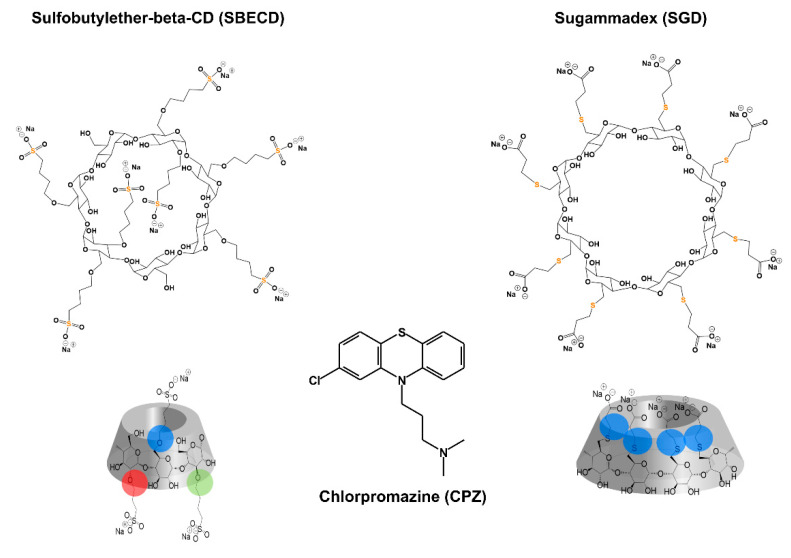
Chemical structures of sulfobutylether-β-cyclodextrin (SBECD), chlorpromazine (CPZ), and sugammadex (SGD), highlighting the location of substituents on the CD scaffolds (red, green and blue circles mark positions 2, 3 and 6, respectively).

Chlorpromazine (CPZ; marketed under brand names such as Thorazine^®^ and Largactil^®^) is an antipsychotic drug with a phenothiazine structure (Figure 1), which can be administered both perorally and parenterally [1]. CPZ was the first antipsychotic drug on the market developed in 1950, and even nowadays it is a widely applied medication which appears on the World Health Organization’s List of Essential Medicines [1,2,3]. It is employed in the pharmacotherapy of schizophrenia and other psychoses, bipolar disorders, and attention deficit hyperactivity disorder (ADHD) [1,4,5]. The antiviral activity of CPZ has also been reported; therefore, its potential application is under investigation in the treatment of COVID-19 and other viral infections [6,7]. During the biotransformation of CPZ, both active and inactive metabolites are formed; however, it is mainly the parent compound that is responsible for the pharmacological activity [1]. CPZ can induce several adverse effects (including sedation, involuntary muscle movements, and prolongation of the QT interval), and can cause severe intoxication at high doses [8]. Acute CPZ poisoning can be treated symptomatically, while no specific antidote is available.

Cyclodextrins (CDs) are ring-shaped molecules built up from glucose units. The most commonly applied CDs are α-, β-, and γ-CDs, which are built up from six, seven, and eight glucopyranose molecules, respectively. The interior cavity of CDs is apolar and can accommodate lipophilic parts of guest molecules; while the hydrophilic exterior space provides excellent aqueous solubility to CDs due to the orientation of hydroxyl groups to their outer surface [9]. CD technology is widely applied by analytical chemistry as well as by food, cosmetic, and pharmaceutical industries, because the microencapsulation of a guest molecule can lead to its solubilization in aqueous environment, improved physicochemical stability, more effective analytical separation or more sensitive instrumental detection, and/or better drug absorption and penetration [10,11,12]. Several suitable complexation techniques can be applied during the preparation of ligand–CD complexes, including complex formation in suspension (CDs and guest substances are dispersed in water during intense agitation then the water is removed by spray-drying or freeze drying, or the complex is filtered and dried), mechanochemical activation (co-grinding of CDs and guest substances with a small amount of water then drying), microencapsulation in water with co-solvent (e.g., ethanol, isopropanol, or glycols; at elevated temperature to obtain a common solution, which is slowly cooled to room temperature, while the complex is precipitated and then filtered and dried), or supercritical carbon dioxide-assisted processes such as the Supercritical AntiSolvent method [13,14,15].

The formation of low-affinity host–guest-type CD complexes typically improves the absorption/penetration of drugs through biological membranes; however, the highly stable ligand–CD complexes can cause the efficient and relatively selective entrapment of the guest molecule in the CD cavity, and can consequently lead to the decreased diffusibility, limited cellular uptake, and/or the more rapid excretion from the body [16,17,18,19]. Based on these principles, CDs can be used to decrease the unpleasant actions of certain drugs and xenobiotics. For example, in the human pharmacotherapy, sugammadex (SGD, a chemically modified γ-CD; Figure 1) is applied for the rapid termination of rocuronium- or vecuronium-induced skeletal muscle relaxation [20]. Since the binding constant (*K*) of rocuronium-SGD and vecuronium-SGD complexes are very high (*K* ≈ 10^7^ L/mol) [21], SGD can effectively compete with nicotinic acetylcholine receptors (the therapeutic target of skeletal muscle relaxants) for ligand binding, leading to the strongly decreased receptorial action of these drugs. Furthermore, hydroxypropyl-β-CD (HPBCD) is an investigational drug in the treatment of Niemann–Pick C disease, due to its interaction with cholesterol [22]. Niemann–Pick C disease is an autosomal recessive lysosomal storage disorder, which is associated with the accumulation of intracellular unesterified cholesterol [23]. In regard to cholesterol, the binding constants of native β-CD, HPBCD, and dimethyl-β-CD are 2 × 10^3^ L/mol, 2 × 10^4^ L/mol and 6 × 10^5^ L/mol, respectively [24]. These data demonstrate that dimethyl-β-CD binds cholesterol with much higher affinity than HPBCD. However, methyl-CDs are relatively toxic CD derivatives and therefore they are not used in human pharmacotherapy, while methyl-CDs are typically applied in certain in vitro experiments for the extraction of cholesterol from the lipid rafts of cell membranes [25]. Importantly, the intravenous (i.v.) administration of some CDs is not recommended (e.g., native β-CD causes nephrotoxicity, or methyl-CDs induce hemolysis); however, other CDs such as HPBCD, sulfobutylether-β-CD (SBECD; Figure 1), and SGD can be administered even parenterally with good tolerability [26,27,28,29]. In addition, a recent patent (US Patent, US 10,442,871 B2; 2019) highlights that certain chemically modified CDs may be suitable for the selective sequestration of fentanyl related compounds.

Furthermore, other studies performed on cell cultures and/or on zebrafish highlighted that CDs are able to relieve the toxic effects of certain xenobiotics due to their entrapment in the CD cavity, including mycotoxins zearalenone [18,19] and alternariol [30], the plant neurotoxin veratridine [31], estradiol [32], perfluorooctanoic acid [33], and compound K (20(S)-protopanaxadiol 20-*O*-D-glucopyranoside) [34]. The binding constants of SBECD, methyl-β-CD, and succinyl–methyl-β-CD complexes with zearalenone were in the range of 1 × 10^4^–5 × 10^4^ L/mol, and alternariol also formed stable complex with SGD (*K* = 5 × 10^4^ L/mol), resulted in the strong protective effects CDs listed against the toxic impacts of these mycotoxins both in HeLa cells and in zebrafish embryos [19,30]. Furthermore, in Neuro-2a cells, SBECD and γ-CD considerably decreased the veratridine-induced loss of cell viability; the *K* values of these toxin–CD complexes were close to 10^4^ L/mol [31]. Estradiol and perfluorooctanoic acid form highly stable complexes with β-CD (*K* = 4 × 10^5^ L/mol and 5 × 10^5^ L/mol, respectively) [35,36], explaining the protective effects of β-CD vs. estradiol- and perfluorooctanoic acid-induced toxicity in zebrafish [32]. Furthermore, complexation of compound K with β-CD not only enhanced its antidiabetic activity but also reduced its toxicity in zebrafish [34]. In a previous study, the interaction of compound K was examined with β- and γ-CDs, where 8 × 10^3^ L/mol has been reported as the binding constant of compound K–β-CD complex [37]. We have not enough available data yet to clearly establish how large binding constants of ligand–CD complexes are required to apply CDs as potential antidotes. However, the above-listed data suggest that approximately 10^4^ L/mol or higher binding constants may give a good starting point.

As has been demonstrated in both in vitro and in vivo experiments, native β- and γ-CDs successfully alleviated the CPZ-induced hemolysis (in vitro) [38], skin irritation [39], and local tissue damage [40,41]. Importantly, in these studies, CPZ–CD complexes were added locally. Thus, there is no available in vivo data in regard to the separate administration of CPZ and CDs, which would be important if we aim to apply CDs as antidotes in CPZ intoxication. In a recent report, the interactions of CPZ were characterized with native and chemically modified CDs [42]. Based on these data, the native β-CD (*K* = 2 × 10^4^ L/mol for 1:1 complexes) forms more stable complexes with CPZ than the native γ-CD (*K* = 5 × 10^2^ L/mol and 6 × 10^3^ L/mol for 1:1 and 1:2 complexes, respectively). Furthermore, the binding constants of the 1:1 complex of CPZ with SBECD and the 1:2 complex of CPZ with SGD were 2 × 10^4^ L/mol and 6 × 10^9^ L/mol, respectively [42]. Considering the highly stable complexes of SBECD and SGD with CPZ as well as the good in vivo tolerability of these CDs even if they are administered parenterally, we felt that the potential antidotal effects of SBECD and SGD against CPZ-induced acute toxicity should be examined. SBECD is a well-defined mixture of isomers (which is also represented in European and US pharmacopoeias), while SGD is a single isomer.

In this study, the impacts of SBECD and SGD were investigated on CPZ-induced acute toxicity. In our in vitro experiments, the protective effects of CDs vs. the CPZ-induced loss of cell viability were tested in the SH-SY5Y neuroblastoma cell line. In addition, the protective action of CDs on CPZ-induced mortality were also examined in vivo, in NMRI mice. Importantly, we did not make directly CPZ–CD inclusion complexes, but we separately added the CD solutions in order to examine their potential antidotal effects. Under these conditions, the dissolved CPZ and CD molecules spontaneously formed inclusion complexes in the cell culture medium and in the body fluids of the animals. Therefore, our results demonstrate that not only the simultaneously and locally added CPZ–CD complex, but the separately applied/administered CDs can also affect the toxic impacts of this antipsychotic drug.

## 2. Materials and Methods

### 2.1. Reagents

Chlorpromazine hydrochloride (CPZ; purity > 98%) was purchased from Henan Tianfu Chemical Co., Ltd. (Zhengzhou, China). Sulfobutylether-β-CD (SBECD; pharmaceutical-grade EP and USP/NF conform quality; purity: 99%; isomeric mixture, complies USP-NF requirements) and sugammadex (SGD; single-isomer γ-CD derivative with a chemical purity >98%; identification and purity determination by NMR, IR, HPLC, and HPLC-MS) were provided by CycloLab Cyclodextrin Research & Development Laboratory, Ltd. (Budapest, Hungary). Dulbecco’s Modified Eagle Medium (DMEM) and fluorescamine (Merck, Darmstadt, Germany), fetal bovine serum (FBS; Pan-Biotech, Aidenbach, Germany), bovine serum albumin (Biosera, Nuaille, France), and bioluminescent ATP Assay Kit CLSII (Roche, Basel, Switzerland) were used as received. All other reagents and solvents applied were of analytical grade.

### 2.2. Cell Experiments

The SH-SY5Y cell line (neuroblastoma, human; ATCC: CRL-2266) was maintained in DMEM supplemented with 10% FBS, penicillin (100 U/mL) and streptomycin (100 µg/mL) and incubated at 37 °C in a humidified environment with 5% CO_2_. Cells were trypsinized then transferred to 96-well plates (10^4^ cells/well). Next day, the medium has been replaced and cells were treated for 24 h with CPZ (0–100 μM), with CDs (0–1500 μM), or with the combinations of CPZ and CDs (final volume: 200 μL/well). After 24 h treatment of cells with CPZ and/or CDs, cells were washed three times with 200 μL of PBS (pH 7.4; also containing 0.18 g/L CaCl_2_, and 0.2 g/L MgCl_2_). Since cell death results in the detachment of cells from the plate, these floating cells and cell debris were removed from the well during these washing steps. Cells were lysed with 200 μL of borate buffer (0.2 M, pH 9.2) containing Triton X-100 (0.1%) detergent and ethylenediaminetetraacetic acid (EDTA, 20 mM), then the plate was placed in a shaker for 5 min. These lysates were applied in cellular ATP and total protein analyses.

The changes in cell viability were primarily evaluated based on ATP levels. ATP content is an indicator of metabolically active cells; therefore, cellular ATP concentration is a suitable parameter to assess the number of viable cells [43,44]. ATP levels were quantified based on the luciferin-luciferase reaction adapted for microplate method, as has been described earlier [45,46]. To quantify cellular ATP concentrations, a 10 μL/well volume of lysates was transferred into white 96-well optical plates (VWR, Debrecen, Hungary), after which 100 μL/well of the dissolved ATP reagent was added. Luminescence data of ATP standards and samples were measured with 5 s integration time, employing an Enspire Multimode reader (Perkin Elmer, Waltham, MA, US).

Since certain compounds can modify cellular ATP levels (which can disrupt the correlation of ATP concentration with the number of living cells) [45,47], total protein measurements were also performed to confirm the ATP-based results. Total protein levels were determined with fluorescamine (Fluram) reagent, as has been previously reported [45,46]. A 20 μL/well volume of the lysed samples were transferred into 96-well plastic plates (VWR, Debrecen, Hungary), after which 150 μL/well and 50 μL/well volumes were added from the lysis buffer (see above) and fluorescamine solution (0.3 mg/mL, in acetone), respectively. After homogenization, the fluorescence signals of these samples were determined with Enspire Multimode plate reader (PerkinElmer) employing 385 nm and 490 nm as excitation and emission wavelengths, respectively. Total protein concentrations were determined based on bovine serum albumin calibration curve.

IC_50_ values were determined by sigmoidal fitting (Hill1) employing the Origin software (OriginLab Corporation, Northampton, MA, US). Statistical analyses were performed employing one-way ANOVA (with Tukey post hoc) test using the IBM SPSS Statistics software (Armonk, NY, US), where the level of significance was set as *p* < 0.01.

### 2.3. Animal Experiments

Female NMRI mice weighing 25–35 g were used for the experiments. Animals were kept in the Laboratory Animal House of the Department of Pharmacology and Pharmacotherapy (University of Pécs) under standard pathogen-free conditions, and were provided with food pellets and water ad libitum. Mice were weighed then treated intraperitoneally (i.p.) with CPZ (120–300 mg/kg, 10 mL/kg, dissolved in physiological saline). Since CPZ typically provoked convulsions after 2 min, SBECD or SGD (500 or 2000 mg/kg, 10 mL/kg, dissolved in physiological saline) was administered i.v. immediately after the CPZ treatment. Control animals were treated with physiological saline (10 mL/kg i.p. and/or i.v.). Before and after the treatment, mice could consume feed and water ad libitum.

We decided to administer CPZ intraperitoneally because it leads to the more rapid appearance of the drug in the systemic circulation compared to the per os treatment. Furthermore, after per os administration, the differences in gastrointestinal absorption typically results in higher variations in plasma concentrations, and usually shows large interspecies differences. On the other hand, we also did not see the i.v. administration of CPZ as advantageous because we wanted to avoid the very rapid development of toxic effects; and with the i.p. treatment, we did not need to stab the tail vein of the mice twice during a very short time period. However, we applied CDs (SBECD or SGD) intravenously to produce immediately their high plasma concentrations as well as to avoid any potential local interactions of CDs with CPZ in the abdominal cavity. Therefore, the potential antidotal impacts of CDs vs. acute CPZ intoxication could be demonstrated with a real separate administration.

As previous studies suggest, female mice are generally more sensitive [48]; therefore, female NMRI mice were applied to determine the LD_50_ values based on 24 h mortality. In agreement with the 3Rs, the up-and-down method was used [48,49,50]. One animal is treated at a time, starting around the LD_50_ value estimated. If the animal survived, then the following animal was treated with a higher dose. If the animal died, then the following animal was treated with a lower dose. This method provides a good estimation of LD_50_, while the number of animals sacrificed can be minimized [48,50,51]. LD_50_ values were determined with the Probit Analysis (CI = 95%) as has been reported [49,50].

This study was performed in agreement with the European legislation (Directive 2010/63/EU) and Hungarian Government regulation (40/2013., II. 14.) in regard to the protection of animals used for scientific purposes. The experiments were approved by the Ethics Committee on Animal Research of University of Pécs (license No.: BA02/2000–05/2021.). A total number of 111 female NMRI mice were used in the experiments.

## 3. Results

### 3.1. Effects of CDs on CPZ-Induced Decrease in Cell Viability

**Figure 2:**
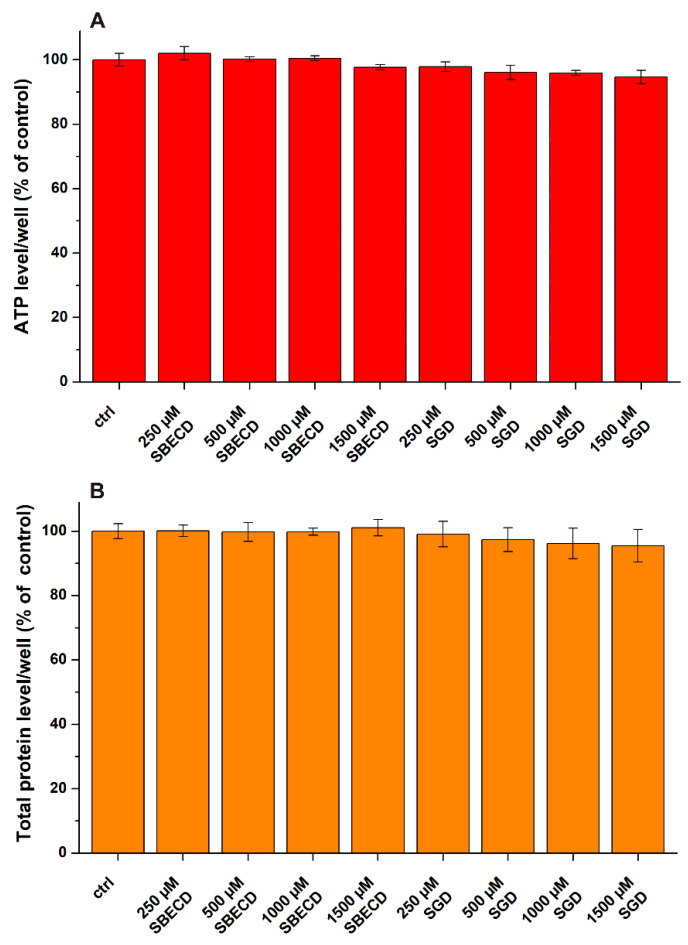
Effects of SBECD and SGD on cellular ATP (**A**) and total protein (**B**) concentrations. SH-SY5Y cells were treated with CDs (0–1500 μM) for 24 h. Data represent the means ± SEM (*n* = 3).

To establish a proper experimental design for co-treatments, we tested first the individual effects of CPZ and CDs on SH-SY5Y cells. Cell viability was primarily evaluated based on ATP concentrations/well. Nevertheless, sometimes the measurement of only one parameter can provide misleading data [45,47]. Therefore, total protein levels/well were also monitored to confirm the results. SBECD and SGD alone did not affect cell viability even at 1500 μM concentration (Figure 2).

**Figure 3:**
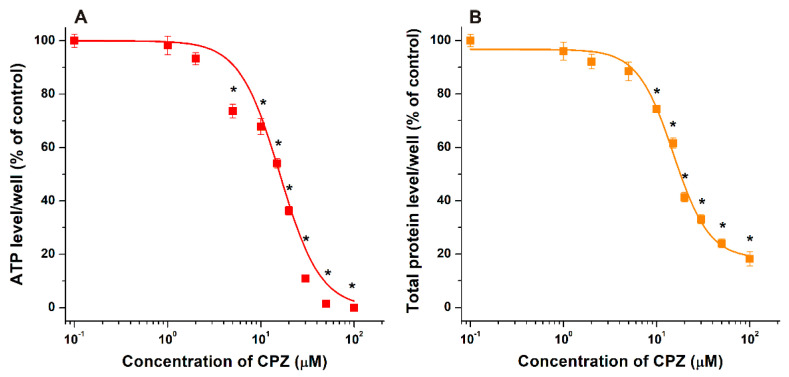
Effects of increasing concentrations of CPZ (0.10, 1.0, 2.0, 5.0, 10, 15, 20, 30, 50, and 100 μM) on the cellular ATP (**A**) and total protein (**B**) levels of SH-SY5Y cells after 24 h incubation. Means ± SEM values are demonstrated (*n* = 3, * *p* < 0.01; see further experimental details in Section 2.2).

However, in a concentration-dependent fashion (the 0–100 μM range has been tested), CPZ strongly decreased ATP (Figure 3A) and total protein (Figure 3B) levels/well in neuroblastoma cells, causing statistically significant (*p* < 0.01) impacts even at 5 μM concentration and showing close the maximal toxicity at 50 μM (Figure 3). Based on the good correlations in regard to the relative changes of ATP and total protein levels, these results demonstrate that CPZ induced considerable loss of cell viability in SH-SY5Y cells at the concentration range applied. Using these data, IC_50_ were determined based on sigmoidal fitting, where 16.0 μM and 15.1 μM values were calculated based on ATP and total protein levels, respectively. Considering the data listed, we selected 20 μM CPZ concentration for the co-treatment experiments which induced approximately 60% decrease in both ATP (Figure 3A) and total protein (Figure 3B) levels.

**Figure 4:**
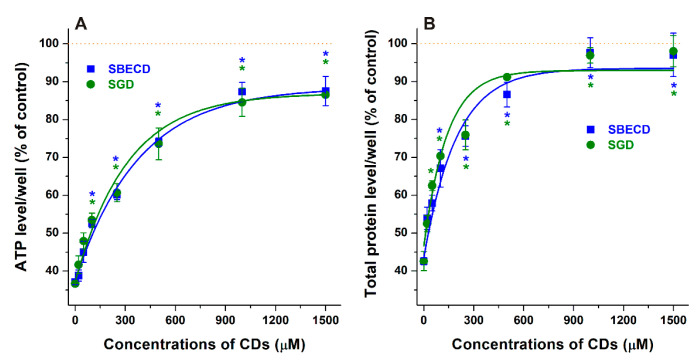
Co-treatment of SH-SY5Y cells with CPZ (20 μM) and CDs (0, 20, 50, 100, 250, 500, 1000, and 1500 μM) for 24 h: SBECD and SGD strongly alleviated the CPZ-induced loss in cell viability based on both cellular ATP (**A**) and total protein (**B**) levels. Means ± SEM values are demonstrated (*n* = 3, * *p* < 0.01; see further experimental details in Section 2.2).

Thereafter, to test the protective effects of SBECD and SGD, cells were simultaneously treated with CPZ (20 μM) and CDs (0–1500 μM). Based on ATP levels, even 100 μM concentrations of SBECD and SGD significantly (*p* < 0.01) increased the viability of CPZ-treated cells (Figure 4A). In addition, higher amounts of CDs (1000 μM and 1500 μM) restored the ATP concentrations to 85–90%. Again, total protein data were in good agreement with the ATP-based results (Figure 4B). Total protein levels showed the slightly stronger impact of SGD which produced statistically significant impact (*p* < 0.01) even at 50 μM concentration, while SBECD induced protective impact from 100 μM. Furthermore, total protein levels were almost completely restored (97–98%) in the presence of 1000 μM and 1500 μM concentrations of SBECD or SGD. Thus, SBECD and SGD showed similarly strong in vitro protective effects against CPZ-induced toxicity.

### 3.2. Effects of CDs on CPZ-Induced 24 h Mortality in Female NMRI Mice

In previously reported studies, the i.p. LD_50_ values of CPZ were in the 150–220 mg/kg range in mice [52,53]. Therefore, we started to treat female NMRI mice with 200 mg/kg CPZ intraperitoneally, after which 500 or 2000 mg/kg doses of CDs were administered intravenously (or physiological saline to the control animals). Then, we followed the up-and-down method depending on the outcome (death of survival). Previous reports suggest that the experimental animals typically dye in the first day of exposure [48], and our aim was to test the suitability of SBECD and SGD as antidotes in acute CPZ intoxication. Therefore, the mortality was evaluated after 24 h. Furthermore, we observed with a few exceptions that CPZ caused its lethal impact typically in the first 2 h. The effects of physiological saline (10 mL/kg i.p. then 10 mL/kg i.v.) as well as the impacts of SBECD and SGD (2000 mg/kg was added i.v. after the i.p. administration of 10 mL/kg physiological saline) were also tested. No mortality was observed in these control groups (*n* = 5). 

**Figure 5:**
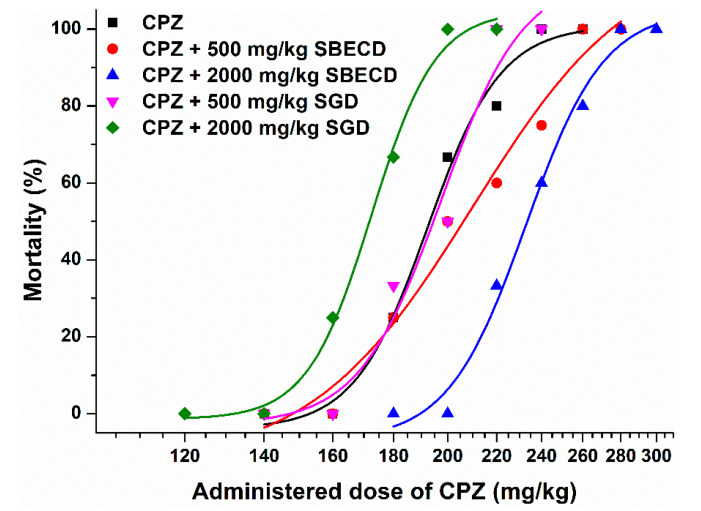
CPZ-induced 24 h mortality without and with SBECD or SGD co-treatment. NMRI mice were treated with CPZ (i.p. 120–300 mg/kg), after which SBECD or SGD (i.v. 500 or 2000 mg/kg; or physiological saline in control animals) was administered (see Section 2.3.).

Figure 5 demonstrates the sigmoidal dose–mortality curves. Interestingly, the lower dose (500 mg/kg) of SGD barely modified the curve. Furthermore, the higher dose (2000 mg/kg) of SGD caused a left shift in the dose–mortality curve, suggesting the increased toxicity of CPZ as a result of this co-treatment. On the other hand, the lower (500 mg/kg) and higher (2000 mg/kg) doses of SBECD led to a slight and a considerable right shift of the curve, respectively (Figure 5). These observations demonstrated the dose-dependent protective action of SBECD vs. the CPZ-induced acute toxicity. Based on the 24 h mortality of CPZ-treated animals, LD_50_ data were determined with the Probit Analysis. In accordance with the visual changes in the dose–mortality curves, the LD_50_ value of CPZ was decreased by SGD (2000 mg/kg) as well as it was slightly and considerably elevated by 500 and 2000 mg/kg doses of SBECD, respectively (Table 1).

## 4. Discussion

Few earlier reports suggest the protective effects of native β-CD and γ-CD vs. the toxic impacts of CPZ [38,39,40,54]. Based on a recent study, SBECD and SGD form highly stable complexes with CPZ [42]. Considering these data and the good in vivo tolerability of SBECD and SGD [20,28], these CDs seemed to be worthy to test their protective actions against CPZ-induced acute toxicity.

In SH-SY5Y cells, the 5 to 100 μM concentrations of CPZ induced a significant decrease in cell viability based on both cellular ATP and total protein data (Figure 3). In agreement with our results, previous reports also suggest the significant toxic effects of CPZ in the 10–60 μM concentration range in several different cell lines [55,56,57]. SBECD and SGD can bind CPZ with high affinity; therefore, the entrapment of CPZ in the CD cavity can limit the cellular uptake and consequently the toxic impacts of the antipsychotic drug, explaining why these CDs were able to relieve the CPZ-induced in vitro toxicity. SGD and SBECD showed similarly strong protective action in our cell experiments (Figure 4). It is surprising because, based on a previous study [42], the binding constant of CPZ–SGD is much higher compared to the CPZ–SBECD complex. The similar impacts of these CDs in cell culture suggest that the difference in the affinity of SBECD and SGD toward CPZ is much lower than it was suggested, and/or SGD may interact with certain compounds in the cell medium or in the cell membranes which can interfere with the formation of CPZ–SGD complexes. In a previous study, native β- and γ-CDs decreased CPZ-induced hemolysis in vitro due to the lower uptake of the drug into erythrocytes [38]. Furthermore, heptakis(2,6-di-O-methyl)-β-CD, β-CD, and γ-CD successfully relieved photosensitized skin irritation caused by CPZ in guinea pigs [39,54]. The intramuscular (i.m.) co-administration of β-CD with CPZ decreased the local tissue damage in rabbits [40]. However, the simultaneous i.m. administration of β-CD (32 mg/kg) and CPZ (10 mg/kg) did not influence the time-course or the magnitude of the CPZ-induced effects (e.g., sedation and suppressed locomotor function) in rats [38]. Interestingly, in another study, SBECD was applied in the formulation of a CPZ-containing osmotic pump tablet, serving as solubilizer and osmotic agent as well as ameliorating the pH-dependence of CPZ release [58].

In previous studies, the i.p. LD_50_ of CPZ was approximately 150–220 mg/kg in mice [52,53], which is in good agreement with our results (Table 1). Based on the in vitro observations in cell experiments (Figure 4), we expected similar effects of SBECD and SGD in animal studies. However, CDs tested caused opposite results in NMRI mice (Figure 5). The lower dose of SGD (500 mg/kg) did not affect, while its higher dose (2000 mg/kg) induced a left shift in the dose–mortality curve of CPZ, showing that even lower doses of the antipsychotic drug caused mortality when mice were co-treated with 2000 mg/kg of SGD. Thus, the higher dose of SGD aggravated the CPZ-induced mortality, leading to a 16 mg/kg decrease in the LD_50_ value of CPZ (Table 1). Similar to this observation, CDs typically increased the caffeine-induced toxicity in zebrafish embryos [59]. Since CDs did not cause the elevated concentrations of caffeine in zebrafish embryos, the higher toxicity of caffeine in the presence of CDs was likely resulted from their synergistic toxic effects. It is important to note that caffeine forms poorly stable complexes with CDs, the binding constant of caffeine–β-CD complex is approximately 10^2^ L/mol [60].

On the other hand, the co-treatment with lower (500 mg/kg) and higher (2000 mg/kg) doses of SBECD caused a slight and a marked right shift of the dose–mortality curve of CPZ, respectively (Figure 5). These observations demonstrate that only higher amounts of CPZ caused mortality when mice were co-treated with SBECD. In a dose-dependent fashion, SBECD increased the LD_50_ values of CPZ, resulting in approximately 10 and 40 mg/kg higher LD_50_ of the antipsychotic drug when mice were co-treated with 500 and 2000 mg/kg SBECD, respectively. Importantly, in previously reported studies [38,39,40,41], cells or animals were treated with CPZ–CD complexes, while our in vivo study demonstrated that the separate administration of CDs can also affect the CPZ-induced toxicity. These observations suggest a clear protective effect of SBECD; however, the relative (1.2-fold) increase in the LD_50_ value is not so large. Therefore, the clinical suitability of SBECD as an antidote of acute, life-threatening CPZ intoxication is questionable.

As has been detailed in the introduction section, the complex stability and/or in vitro cell experiments can help to make a prediction in regard to the in vivo effects. In some studies, the binding affinity of ligand–CD complexes showed excellent correlation with the in vitro and/or in vivo protective actions of CDs [19,32,33]. However, sometimes other factors can overwrite these expectations [18,30]. A recent study showed that mycotoxin alternariol forms highly stable complex with SGD (*K* = 5 × 10^4^ L/mol), while the stability of alternariol-SBECD (*K* = 2 × 10^3^ L/mol) and alternariol–β-CD (*K* = 3 × 10^2^ L/mol) complexes were considerably lower [30,61]. In agreement with these data, β-CD did not affect, SBECD slightly relieved, while SGD markedly alleviated the alternariol-induced cytotoxicity in HeLa cells [30]. In contrast, in the in vivo zebrafish study, each CD showed strong protective effects against the toxic action of alternariol, and importantly native β-CD decreased most successfully the alternariol-induced mortality and malformations [30]. Furthermore, in animal experiments, controversial results have been reported in regard to verapamil-CD co-treatment as well. SBECD (2.25 g/kg) aggravated the verapamil-induced (32 mg/kg/h) toxicity in rats [62]. Interestingly, co-treatment of rats with verapamil infusion (32 mg/kg/h) and 4-fold concentration of SBECD resulted in the slightly prolonged time to asystole compared to the control, while the higher or lower amounts of SBECD did not cause significant impacts [63]. In another study, the low dose of SGD (16 mg/kg) delayed verapamil (37.5 mg/kg/h) cardiotoxicity, while its high dose (1000 mg/kg) accelerated it in rats [64]. The above-listed results demonstrate that the in vivo action of CDs as toxin binders and/or their applications as antidotes in certain drug intoxications are highly complicated. Besides the stability of ligand–CD complexes, other, still unknown, parameters can affect their potential utilization.

In summary, this is the first study where: (1) the impacts of the SBECD and SGD were examined vs. the toxic effects of CPZ; (2) the in vivo antidotal/protective actions of CDs were tested with separate administration of CPZ and CDs; (3) the impacts of CDs were investigated in vivo thorough their protective actions vs. lethal CPZ intoxication, as a hard endpoint. Furthermore, typically the impacts of CDs on the toxic effects of certain compounds are examined employing in vitro or in vivo models [18,31,33,34], while we made an in vitro vs. in vivo comparison. Both SBECD and SGD showed similarly strong protective impacts in SH-SY5Y cells against the CPZ-induced toxicity. Unexpectedly, despite the very high affinity of CPZ–SGD complexes and the strong protective action of SGD in cell experiments, SGD co-treatment did not affect (500 mg/kg) or even increased (2000 mg/kg) the CPZ-induced mortality in NMRI mice. In a dose-dependent fashion, SBECD alleviated CPZ-induced loss of cell viability in cell experiments and also decreased the CPZ-induced mortality in animal studies. Nevertheless, the in vivo protective action of CPZ was lower than we expected based on the in vitro studies. Our results demonstrate that the separate administration of CDs can also modify the acute toxic impacts of CPZ. However, based on the marked differences between our in vitro and in vivo observations, it is difficult to predict the in vivo suitability of CDs as antidotes based on the binding constants of ligand–CD complexes and/or the effects of CDs in cell experiments. Therefore, further extensive in vivo studies are reasonable for the deeper understanding of the application of CDs for detoxication purposes. Nevertheless, even if we consider the above-listed difficulties, CDs seem to be promising candidates to bind and remove different toxic compounds from aqueous matrices, and/or to develop new detoxification strategies or antidotes against certain xenobiotics.

## Figures and Tables

**Table 1:** LD_50_ values calculated employing the Probit Analysis based on the 24 h mortality data of female NMRI mice.

	CPZ (Control)	CPZ + 500 mg/kg SBECD	CPZ + 2000 mg/kg SBECD	CPZ + 500 mg/kg SGD	CPZ + 2000 mg/kg SGD
LD_50_ (mg/kg, based on 24 h mortality)	194.0	206.2	232.8	200.0	171.8

## Data Availability

Not applicable.
